# Identification of bilateral fornix and hippocampus in fetuses between 18–36 gestational weeks and establishment of nomograms using ultrasonography

**DOI:** 10.1007/s00404-025-08061-z

**Published:** 2025-05-23

**Authors:** Nazli Ece Sahin, Ebru Alici Davutoglu, Gorkem Arica, Riza Madazli

**Affiliations:** 1 Cerrahpasa Medical Faculty, Department of Obstetrics and Gynecology, Division of Perinatology, Istanbul University-Cerrahpasa, Istanbul, Turkey; 2 Cerrahpasa Medical Faculty, Department of Obstetrics and Gynecology, Istanbul University-Cerrahpasa, Kocamustafapasa, Istanbul, Turkey

**Keywords:** Fetal brain, Fornix, Hippocampus, Nomograms, Neurosonography

## Abstract

**Introduction:**

To establish nomograms for the bilateral fornix-hippocampus complex (FHC) length and hippocampus height (HH) in fetuses between 18 + 0 and 36 + 0 weeks of gestation using two-dimensional (2D) sonography and assess potential laterality differences.

**Methods:**

A prospective study was conducted on 725 singleton pregnancies at the Maternal–Fetal Medicine Department from January to December 2024. Gestational age was confirmed via first-trimester crown-rump length measurements. Exclusion criteria included maternal chronic diseases, multiple pregnancies, fetal anomalies, and growth restriction. Transabdominal 2D sonography captured FHC and HH measurements in the parasagittal plane. Laterality was determined before imaging, and each measurement was repeated twice for reliability.

Percentiles (5th, 50th, and 95th) were calculated, and Pearson correlation coefficients assessed relationships with gestational age. The independent samples t-test compared left and right measurements, and intraobserver reliability was evaluated with the intraclass correlation coefficient (ICC).

**Results:**

Nomograms with 5th, 50th, and 95th percentiles were established for both sides. The Pearson correlation coefficients for the left and right sides of the FHC length and HH with advancing gestational age were 0.808, 0.808, 0.725, and 0.734, respectively. The correlation coefficients between FHC length and HH for the left and right sides were 0.814 and 0.818, respectively. No significant laterality differences were found (*p* > 0.05).

**Discussion:**

These nomograms provide reference data for assessing fetal neurodevelopment. They may aid in detecting abnormalities, particularly in corpus callosum agenesis and ventriculomegaly.

## What does this study add to the clinical work

**Table:** 

This is the first study to establish nomograms for the fetal hippocampus and fornix across a wide range of gestational ages using a large patient cohort. These normative reference values may serve as a valuable resource for future research in detecting abnormalities in these structures.	

## Introduction

The hippocampus is located in the medial portion of the anterior temporal lobe and is in close proximity to the temporal horn of the lateral ventricle [1]. It plays a major role in memory processing and in biological responses to stress [2, 3]. The fornices are two C-shaped structures that extend obliquely from just inferior to the corpus callosum to the hippocampus and include efferent tracts that help maintain their integrity [4]. Given their functional continuity, these two anatomical regions are often evaluated together [5].

The development of the hippocampal formation has been described in the literature using diagrams that illustrate the progressive infolding of the fetal dentate gyrus, cornu ammonis, subiculum, and parahippocampal gyrus around the progressively narrowing hippocampal sulcus (hippocampal fissure) [6–8]. The hippocampal structures begin to fold around the hippocampal sulcus at 15 weeks of gestation, with the hippocampus attaining its adult shape by 18 to 20 weeks [9].

Hippocampal development is closely associated with intracranial structures such as the lateral ventricles, corpus callosum, fornix, and temporal lobe. Abnormal hippocampus-fornix formation has been linked to congenital brain anomalies such as agenesis of the corpus callosum, lissencephaly, and holoprosencephaly [10]. Additionally, incomplete hippocampal inversion has been observed with increased frequency in neurodevelopmental disorders, schizophrenia, and epilepsy [11–15].

Examination of the fetal hippocampus and fornix is not a routine part of fetal neurosonography. Moreover, two-dimensional sonographic imaging of the fetal hippocampus and fornix is challenging due to their curved, oblique C-shaped structure, with the upper part positioned medially and the lower part laterally. There are limited studies in the literature on ultrasonographic imaging of the fetal hippocampus and fornix. In this study, we developed growth nomograms for the fetal fornix-hippocampus complex (FHC) length and hippocampus height (HH) on both sides between 18 + 0 and 36 + 0 weeks of gestation using two-dimensional (2D) sonography. Additionally, we compared these measurements for laterality.

## Methods

This prospective study was conducted at the Maternal–Fetal Medicine Department of the University Hospital between January 2024 and December 2024. The study protocol was approved by the Ethics Committee of our university (Date: 26/01/2024; Number: E-83045809-604.01-897587), and all participants provided written informed consent. The study adhered to the principles of the Declaration of Helsinki.

A total of 725 pregnancies between 18 + 0 and 36 + 0 weeks of gestation, referred to the maternal–fetal medicine department, were enrolled. Gestational age was determined based on the first day of the last menstrual period and confirmed by first-trimester crown-to-rump length measurements. Exclusion criteria included pregestational and gestational diabetes mellitus, chronic diseases, multiple pregnancies, fetuses with known or suspected structural abnormalities, genetic anomalies, fetal growth restriction, and participants who declined to participate.

All scans were performed transabdominally by a maternal–fetal medicine fellow (G.A.), under the supervision of experts (R.M., E.A.D.), using 2D ultrasound in transverse, coronal, sagittal and parasagittal planes. Each measurement was performed twice, with a 2-min interval, to assess intraobserver reliability. The ultrasound equipment included Voluson E10 and Voluson S8 machines (GE Healthcare, Zipf, Austria) with 5- and 3.5-MHz transducers.

First, fetal laterality was determined to ensure accurate left and right side measurements of the fornix and hippocampus. The insonation angle of the ultrasound probe was adjusted in the frontoparietal or occipitoparietal direction to optimize imaging and minimize acoustic shadowing.

**Fig. 1:**
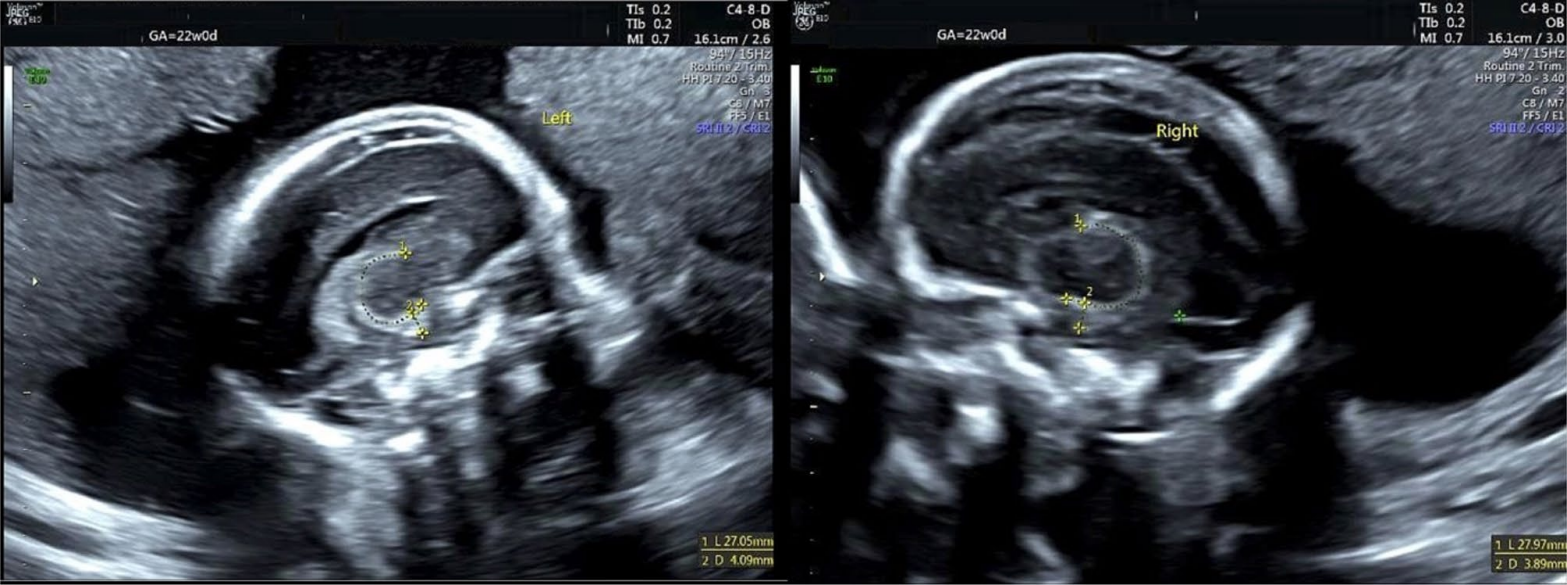
The fetal fornix and hippocampus appear as crescent-shaped hyperechogenic borders surrounding the thalamus. The trace line between the plus marks indicates the length of the fornix and hippocampus. The height of the hippocampus is measured by determining the widest distance between the hyperechogenic lines formed by the choroidal fissure

Initially, the fetal corpus callosum was visualized in a sagittal plane. The probe was then angled laterally to visualize the fetal thalamus. Further lateral angling allowed imaging of the fetal fornix, followed distally by the hippocampus. In this view, the fornix and hippocampus appeared as crescent-shaped hyperechogenic borders surrounding the thalamus (Fig. 1). The total length of the fornix-hippocampus complex (FHC) was measured using the trace mode. As the hippocampus could not be distinguished from the fornix in two-dimensional imaging, volumetric assessment was not feasible. Hippocampal height was measured as the widest distance between the hyperechogenic lines of the choroidal fissure [16]. Measurements were performed in cine mode, duplicated, and averaged. All measurements were performed in a blinded manner.

Before 32 weeks of gestation, we achieved a 100% success rate in visualizing the bilateral FHC in both vertex and breech presentations. However, after 32 weeks, the success rate for identifying the FHC decreased due to the engagement of the fetal head in the pelvis. We included only patients for whom sufficient imaging of both FHC could be obtained, while fetuses with inadequate bilateral assessment of the FHC were excluded. We did not perform transvaginal sonography due to the low acceptance rate of this examination among women in our population.

### Statistical analysis

Data were analyzed using SPSS software version 29.0 (Chicago, IL, USA). Results were expressed as mean, standard deviation, percentages, and numbers and presented in tables according to gestational age and laterality.

Laterality comparisons were conducted using the independent samples t-test. Polynomial regression analysis was performed, and the 5 th, 50 th, and 95 th percentiles for each parameter were plotted against gestational age. Pearson correlation coefficients were calculated, with statistical significance set at *p* ≤ 0.05.

Intraobserver agreement was assessed by comparing the two measurements obtained by the same observer. A two-way random model was used to calculate the single measure intraclass correlation coefficient (ICC) for absolute agreement.

## Results

The 5 th and 95 th percentiles of left and right fetal fornix–hippocampus complex length and hippocampus height are illustrated in Tables 1 and 2. Table 3 presents a comparison of FHC length and HH measurements based on laterality. Notably, no significant differences in FHC length (*p* > 0.05) or HH (*p* > 0.05) were observed between the right and left sides across all gestational age groups.

**Table 1:** 5 th, 50 th, and 95 th percentiles of right and left fetal fornix–hippocampus complex length according to gestational age

GA (weeks)	N	Right fornix–hippocampus complex (mm)			Left fornix–hippocampus complex (mm)		
		5%	50%	95%	5%	50%	95%
18^+0^–19^+6^	53	21.1	25.1	34.1	20.2	25.1	31.1
20^+0^–21^+6^	222	25.2	29.9	34.0	24.9	29.4	34.5
22^+0^–23^+6^	244	28.0	32.1	37.3	27.9	32.0	38.1
24^+0^–25^+6^	53	29.5	35.1	42.3	29.5	36.4	42.3
26^+0^–27^+6^	47	32.0	39.5	49.4	31.9	40.9	50.7
28^+0^–29^+6^	44	37.5	44.8	50.7	39.1	45.6	51.3
30^+0^–31^+6^	22	40.1	45.5	57.9	43.2	45.7	57.9
32^+0^–33^+6^	20	43.2	50.1	58.6	43.3	50.2	58.2
34^+0^–36^+0^	20	44.4	53.4	59.8	47.1	54.0	58.8

**Table 2:** 5 th, 50 th, and 95 th percentiles of right and left hippocampus height according to gestational age

GA (weeks)	N	Right hippocampus height (mm)			Left hippocampus height (mm)		
		5%	50%	95%	5%	50%	95%
18^+0^–19^+6^	53	2.67	3.28	4.41	2.66	3.26	4.30
20^+0^–21^+6^	222	3.07	3.80	4.72	3.10	3.76	4.75
22^+0^–23^+6^	244	3.13	4.09	5.20	3.18	4.10	5.16
24^+0^–25^+6^	53	3.60	5.01	6.03	3.50	4.71	6.11
26^+0^–27^+6^	47	3.93	5.57	7.00	3.91	5.20	7.20
28^+0^–29^+6^	44	5.10	6.10	7.60	5.19	6.16	7.87
30^+0^–31^+6^	22	5.31	6.40	8.04	5.33	6.40	8.13
32^+0^–33^+6^	20	5.63	6.41	8.80	5.52	6.44	8.68
34^+0^–36^+0^	20	6.20	7.25	8.89	5.71	7.30	8.89

**Table 3:** Comparison of fetal fornix–hippocampus complex length and hippocampus height measurements according to laterality

Laterality				Laterality		
Gestational age (weeks)	Right FHC (mm)	Left FHC (mm)	p	Right HH (mm)	Left HH (mm)	p
18^+0^–19^+6^	25.5 ± 3.3	25.4 ± 3.2	0.789	3.36 ± 0.47	3.38 ± 0.45	0.835
20^+0^–21^+6^	29.8 ± 3.1	29.7 ± 3.3	0.729	3.85 ± 0.64	3.84 ± 0.65	0.874
22^+0^–23^+6^	32.4 ± 2.9	32.3 ± 3.1	0.903	4.11 ± 0.57	4.11 ± 0.59	0.746
24^+0^–25^+6^	35.0 ± 3.9	35.7 ± 3.9	0.335	4.76 ± 0.71	4.78 ± 0.67	0.846
26^+0^–27^+6^	39.7 ± 5.0	40.5 ± 5.5	0.409	5.39 ± 0.82	5.25 ± 0.80	0.445
28^+0^–29^+6^	44.9 ± 4.0	45.6 ± 3.3	0.882	6.14 ± 0.71	6.17 ± 0.86	0.364
30^+0^–31^+6^	46.7 ± 4.6	46.9 ± 3.9	0.893	6.49 ± 0.64	6.50 ± 0.67	0.980
32^+0^–33^+6^	49.7 ± 4.9	49.7 ± 4.5	0.992	6.97 ± 1.1	6.92 ± 0.9	0.883
34^+0^–36^+0^	53.5 ± 3.4	53.8 ± 2.8	0.774	7.32 ± 0.7	7.35 ± 0.8	0.888

**Fig. 2:**
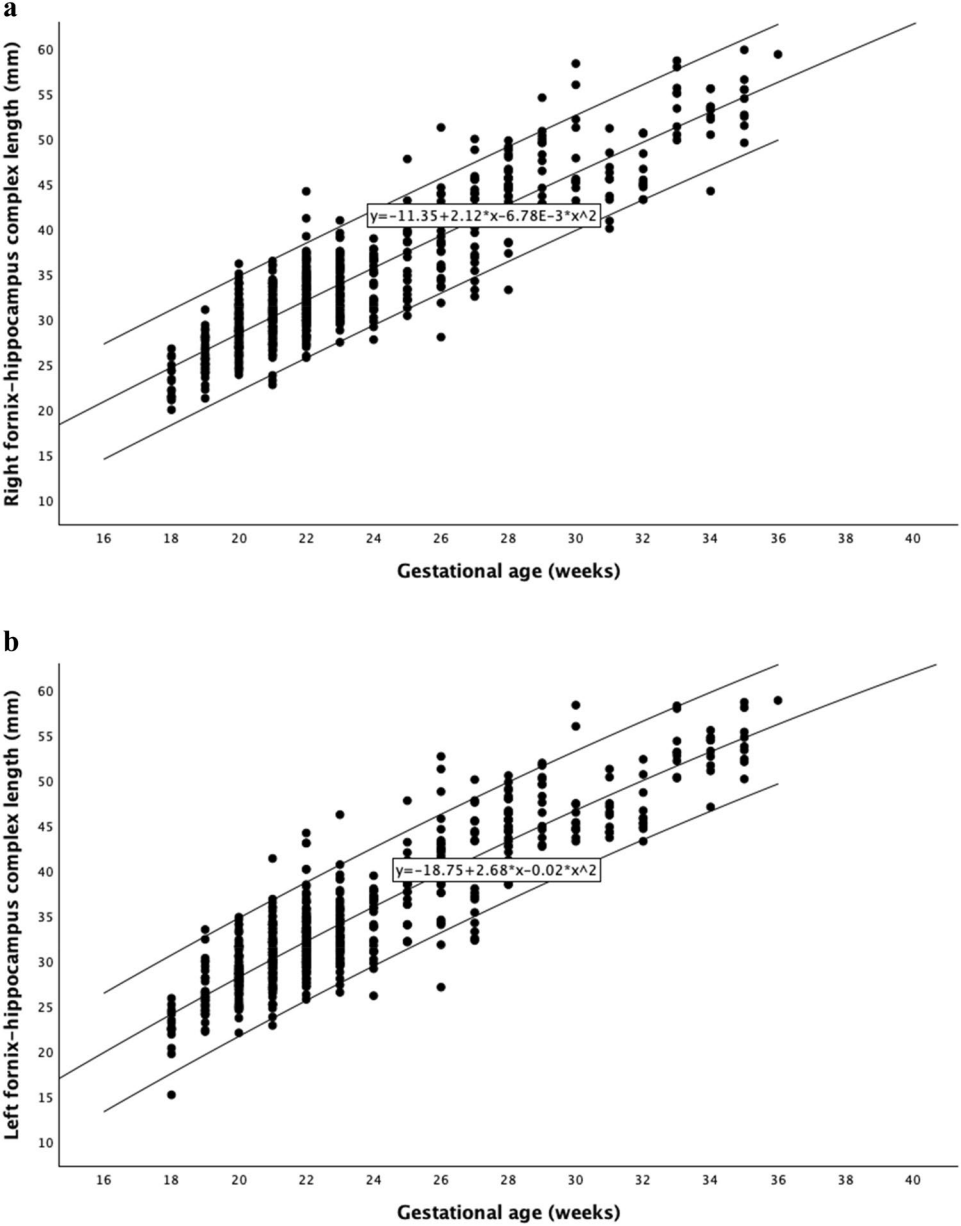
Reference curves for right (**a**) and left (**b**) fornix–hippocampus complex length between 18 and 36 weeks of gestation, with the 5 th, 50 th, and 95 th percentiles

**Fig. 3:**
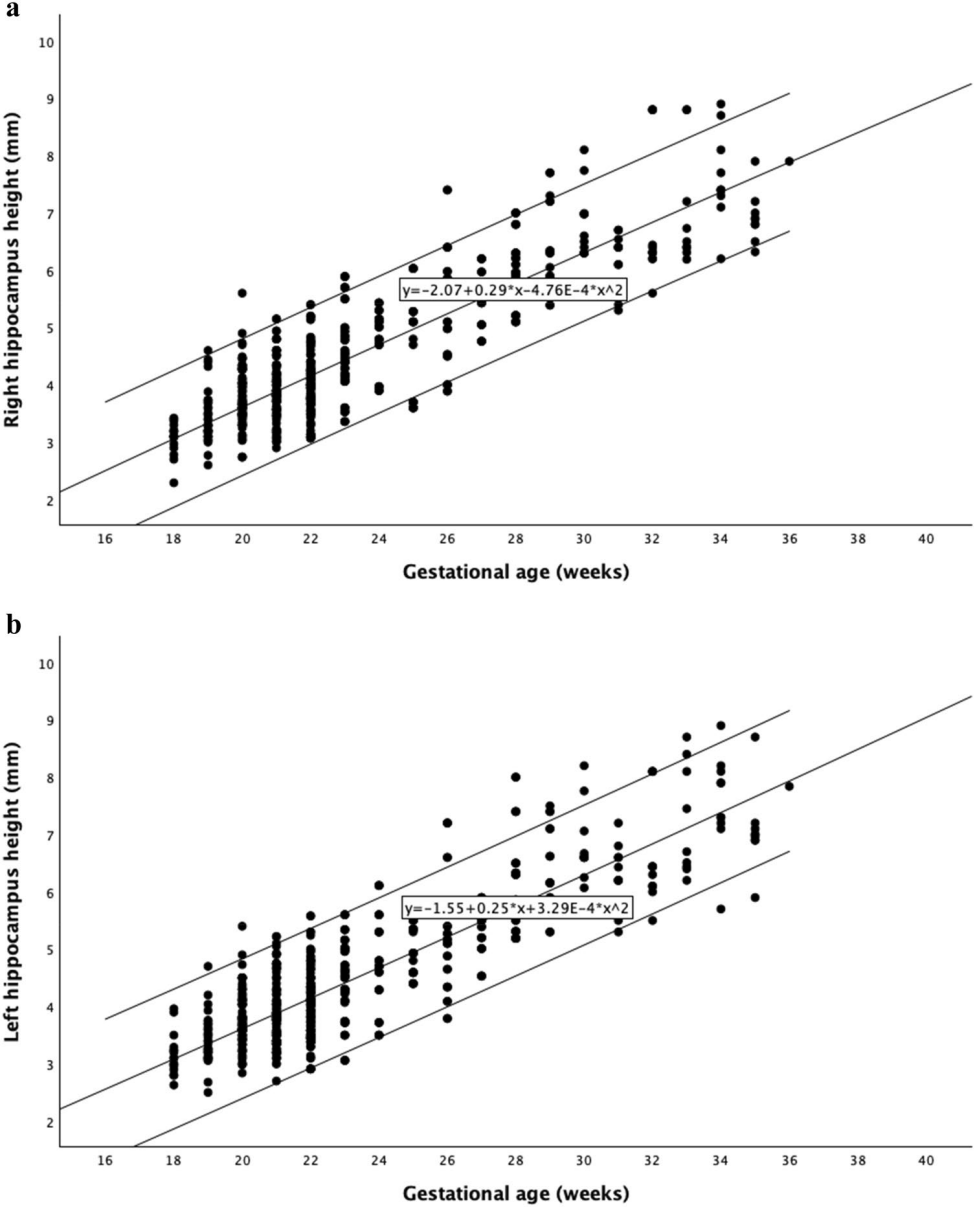
Reference curves for right (**a**) and left (**b**) hippocampus height between 18 and 36 weeks of gestation, with the 5 th, 50 th, and 95 th percentiles

Individual measurements of left and right FHC length and HH in 725 fetuses, along with the derived reference ranges for gestational age including mean, 5 th, and 95 th percentiles are shown in Figs. 2 and 3. There was a significant increase in the fetal FHC length and hippocampus height with advancing gestational age. The Pearson correlation coefficients for the left and right sides of the FHC length and HH with advancing gestational age were 0.808, 0.808, 0.725, and 0.734, respectively. A significant correlation was also observed between fetal FHC length and hippocampus height. The correlation coefficients between FHC length and HH for the left and right sides were 0.814 and 0.818, respectively. To assess potential differences related to fetal sex, measurements from male and female fetuses were compared. The study group consisted of 366 male and 359 female fetuses. No significant differences were found between male and female fetuses for either right or left fetal FHC lengths (*p* > 0.05) or right or left fetal hippocampus heights (*p* > 0.05) across all gestational age groups. The intraobserver ICC was 0.994 (95% CI 0.993–0.995), indicating almost perfect agreement for fetal FHC length and hippocampus height measurements.

## Discussion

In this study, we present our established nomograms for fetal fornix-hippocampus complex length and hippocampus height between 18^+0^ and 36^+0^ weeks of gestation by two-dimensional (2D) sonography. To our knowledge, nomograms for the fetal hippocampus and fornix across a wide range of gestational ages, based on a large patient cohort, have not been previously reported. Toprak et al. measured unilateral FHC length and hippocampus height at 18 and 24 weeks of gestation in 90 fetuses, with measurements comparable to those in our study [16]. Additionally, a 3D ultrasound study by Gindes et al. measured fornix and hippocampus lengths in 32 fetuses between 14 and 37 weeks of gestation [5]. They also assessed differences between the left and right fornix and hippocampus measurements and found no significant differences.

There are inconsistent findings in the literature regarding the development of the right and left sides of the hippocampus and fornix. Righini et al. evaluated the hippocampal infolding angle in fetuses between 20 and 37 weeks of gestation and found no significant differences in the development of both sides of the hippocampus in their prenatal MRI study [17]. Nichols et al. investigated hippocampal development based on laterality from the beginning of the third trimester to four years postnatally [18]. They found that the left hippocampus develops faster than the right from the third trimester to two years of age, after which the right side catches up to the left. Another study by Bajic et al. evaluated hippocampal development using cranial ultrasound in preterm neonates between 23 and 36 weeks of gestation [19]. Their findings indicated that the right hippocampus develops faster than the left, as evidenced by higher rates of impaired infolding on the left side. In the present study, we found no significant differences between the measurements of the two sides of the FHC and hippocampus. Unlike previous studies, we evaluated the hippocampus in the parasagittal plane rather than the coronal plane and measured hippocampal height instead of the infolding angle. These inconsistencies may be attributed to variations in study methodologies and imaging techniques.

The effect of fetal gender on the development of intracranial cortical structures has been evaluated in previous studies. It has been reported that the cortical sulcus depths in male fetuses become significantly greater than in female fetuses after 28 weeks of gestation [20]. Specifically for the hippocampus, a prenatal MRI-based volumetric study conducted by Nichols et al. demonstrated that hippocampal volumes in male fetuses developed more rapidly than those in female fetuses during the prenatal period [18]. In a previous study published by our group, which assessed bilateral fetal FHC length and HH between 18 and 24 weeks of gestation, we found no significant differences in measurements between male and female fetuses [21]. Similarly, in the present study, we did not observe any significant differences based on fetal gender across each gestational week assessed.

Congenital central nervous system (CNS) malformations are a common group of anomalies that have significant clinical importance. These malformations are associated with high rates of morbidity and mortality, which can impact the neurocognitive and motor development of the survivors [22]. As a result, it is crucial to assess the fetal CNS during the prenatal period to identify any changes in its development and provide appropriate guidance to parents regarding pregnancy follow-up, options for termination, postnatal treatment, and prognosis. Multiplanar fetal neurosonography enables detailed anatomic examination of the fetal CNS.

Numerous studies have demonstrated that cerebral malformations, such as agenesis of the corpus callosum, cortical malformation, and holoprosencephaly, are influenced by abnormal hippocampal formation [10, 22]. Additionally, Whitehead et al. observed a correlation between ventriculomegaly and incomplete hippocampal inversion, suggesting that incomplete hippocampal inversion may contribute to lateral ventriculomegaly [23]. Establishing normal reference values for the FHC and hippocampus may be valuable for future research in detecting abnormalities in these structures, particularly in cases of corpus callosum agenesis and ventriculomegaly.

This study has several notable strengths. First, it was designed prospectively, enhancing the quality and reliability of the data collected [24]. The study employed targeted neurosonographic assessment of intracranial structures, using standardized 2D ultrasound protocols in multiple planes. All measurements were performed by a trained operator under expert supervision, ensuring technical consistency. Additionally, the study population consisted of low-risk pregnant women, reducing potential confounding factors. Importantly, the intraobserver reliability was excellent, as demonstrated by a high ICC, supporting the precision of the measurements.

The main limitation of our study is that we measured hippocampal height, which may not accurately reflect the true volume of the hippocampus, particularly in the presence of impairments in hippocampal infolding. Another limitation of the study is that interobserver variability could not be assessed, as all measurements were performed by a single operator, which may limit the reproducibility of the data.

In conclusion, our study demonstrates that bilateral FHC can be visualized with 2D ultrasonography from 18 to 36 gestational week and normal reference values providing valuable additional information in this area.

## Data Availability

The data that support the findings of this study are available from the corresponding author, upon reasonable request.
